# The Role of Th17 in Neuroimmune Disorders: Target for CAM Therapy. Part I

**DOI:** 10.1093/ecam/nep062

**Published:** 2011-06-16

**Authors:** Aristo Vojdani, Jama Lambert

**Affiliations:** Immunosciences Lab., Inc., Los Angeles, CA 90035, USA

## Abstract

CD4^+^ effector cells, based on cytokine production, nuclear receptors and signaling pathways, have been categorized into four subsets. T-helper-1 cells produce IFN-**γ**, TNF-**β**, lymphotoxin and IL-10; T-helper-2 cells produce IL-4, IL-5, IL-10, IL-13, IL-21 and IL-31; T-helper-3, or regulatory T-cells, produce IL-10, TGF-**β** and IL-35; and the recently discovered T-helper-17 cell produces IL-17, IL-17A, IL-17F, IL-21, IL-26 and CCL20. By producing IL-17 and other signaling molecules, Th17 contributes to the pathogenesis of multiple autoimmune diseases including allergic inflammation, rheumatoid arthritis, autoimmune gastritis, inflammatory bowel disease, psoriasis and multiple sclerosis. In this article, we review the differential regulation of inflammation in different tissues with a major emphasis on enhancement of neuroinflammation by local production of IL-17 in the brain. By understanding the role of pathogenic factors in the induction of autoimmune diseases by Th17 cells, CAM practitioners will be able to design CAM therapies targeting Th17 and associated cytokine activities and signaling pathways to repair the intestinal and blood-brain barriers for their patients with autoimmunities, in particular, those with neuroinflammation and neurodegeneration.

## 1. Introduction

**Figure 1:**
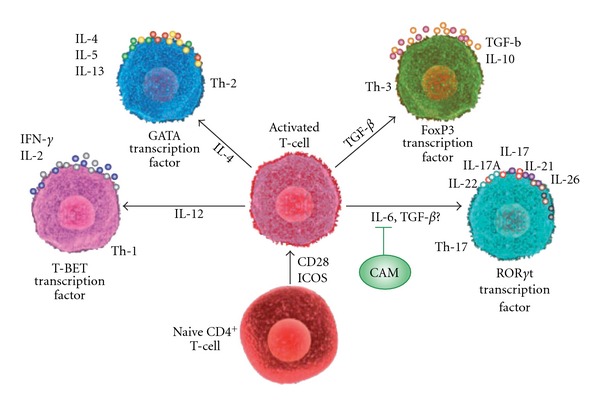
General scheme of T-helper cell differentiation. Naive CD4^+^ T cells, after activation by T-cell receptor and co-stimulatory molecules, such as CD28 and inducible T-cell co-stimulator (ICOS), can differentiate into four effector T-helper cells: Th1, Th2, Th3 or Th17 cells. These cells produce different cytokines, which have specialized immunoregulatory functions. IFN-*γ* produced by Th1 cells is important in the regulation of antigen presentation and cellular immunity. IL-4, IL-5 and IL-13 produced by Th2 cells regulate B-cell responses, important mediators of allergic diseases. TGF-*β* and IL-10 are produced by Th3 cells to regulate Th1 and Th2 cells. Th17 cells regulate inflammatory responses by expressing IL-17, IL-21, IL-22 and IL-26. CAM protocols can be implemented to reduce the level of proinflammatory cytokine IL-6, thereby inhibiting the conversion of activated T cells into pathogenic Th17 cells.

For more than 30 years T-helper (Th) cells have been divided by immunologists into two functional subsets: T-helper-1 (Th1) and T-helper-2 (Th2). Th1 and Th2 subsets are characterized by a distinct activity of transcription factor and pattern of cytokine-secretion phenotype [1, 2]. This differentiation of CD4^+^ T-cells toward Th1, Th2 and other subsets depends on an appropriate signal through the TCR and generated cytokine milieu is an important factor that influences CD4 cell lineage commitment. For example, interleukin (IL)-12 activates STAT4 and drives naïve CD4^+^ T cells to become Th1 cells that produce interferon-gamma (INF-*γ*). These signals from IL-12 and IFN-*γ* by acting through STAT4 and STAT1, increase the expression of the transcription factor called T-bet, which promotes further production of IFN-*γ* and commitment to the Th1 cell lineage. Th1 cells classically produce IFN-*γ*, tumor necrosis factor-beta (TNF-*β*) and interleukin-10 (IL-10), and mediate cellular immune responses against tumor cells, intracellular viruses and bacteria through activation of macrophages and cytotoxic T-cells. In addition, Th1 cells drive cell-mediated response leading to tissue damage and drive humoral immune responses in certain immunoglobulin subclasses termed Ig2a. Innate immune cells, by signals through STAT6, secrete IL-4 that induces naïve CD4^+^ T cells to become Th2 cells. This leads to the expression of transcription factor GATA3. This cascade of events in turn results in the production of IL-4, IL-5, IL-13, IL-21 and IL-31, which are important for host defense against helminths and contribute to the pathogenesis of asthma and allergy [3–7]. Another lineage of T-cells that co-express CD4 and CD25 are Th3 cells, or regulatory T (T_REG_) cells, which have the capacity to regulate both Th1 and Th2 cell function, and maintain homeostasis in the immune system [8, 9]. T_REG_ cells can be developed from thymic CD4^+^ T-cell precursors in the presence of IL-2 and TGF-*β*, which are termed natural T_REG_ cells. In the periphery, naïve CD4^+^ T cells, by signaling through STAT5 in the presence of transforming growth factor-beta (TGF-*β*) can also be converted and become inducible T_REG_, which express transcription factor FoxP3. T_REG_ cells produce low levels of IL-2 and IFN-*γ*, but produce high levels of IL-10, IL-35 and TGF-*β*. T_REG_ cells have an important role in peripheral self-tolerance and immune suppression. This crucial role of T_REG_s in suppressing immune responses to self-antigens and in preventing autoimmune disease is done by two different immunoregulatory immunosuppressive or anti-inflammatory cytokines, IL-10 and TGF-*β*. The importance of the T_REG_ in the modulation of the immune system and its potential for CAM intervention was reviewed by the senior author earlier in this journal [10–12]. In recent years, a specific T-cell subset, termed Th17 cell, with the capacity to produce a distinct cytokine called IL-17, has been identified [13]. Th17 cells develop from naïve CD4^+^ T cells in response to IL-6, IL-23, TGF-*β* and IL-1*β*. IL-6 and IL-23 activate STAT3, which increases the expression of ROR*γ*t and ROR*γ* transcription factors, which in turn promote the expression of IL-17A, IL-17F, IL-21 and IL-22. Th17 cells are important for host defense against extracellular bacteria and are involved in mediating autoimmune diseases [6, 14, 15]. This subdivision of T-helper cells and their differentiation from a naïve CD4 cell is shown in Figure 1. The immunopathogenic activities of Th17 cell in inflammation and autoimmunity with possible CAM intervention is discussed in this review article.

Since the past decade has witnessed many revisions in the Th1/Th2 hypothesis and the involvement of Th1/Th2 in T-cell-mediated tissue damage, it is possible in the future for many additional complexities related to CD4^+^ T-cell diversity to become evident in the field of human immunology. Therefore, CAM researchers should be aware that the extent to which some aspects of T-cell subsets in humans are firmly fixed while others may change will continue to be the subject of intense investigation and future reports.

## 2. Immunopathogenicity of Th17 Cells

The role of Th17 lymphocytes in immunopathogenic processes has recently been formally established. T-cells producing IL-17 constitute a new lineage of CD4^+^ T-cells developed directly from naïve CD4 T-cells, which seems to play key roles in the activation of neutrophils and the immunity to bacteria, particularly at mucosal surfaces. The Th17 cell has also been linked to a growing list of cancers, autoimmune and inflammatory diseases such as rheumatoid arthritis, systemic lupus erythematosus, multiple sclerosis, asthma, psoriasis, systemic sclerosis, chronic inflammatory bowel disease and allograft rejection [6, 13–20].

Although the importance of IL-17-producing CD4^+^ T cells in the pathogenesis of autoimmune diseases is widely accepted, the signaling pathway involved in the development and maintenance of these cells only recently became clear [21–23]. In this model, interleukin-23 (IL-23) plays a significant role in the development of Th17 cells. Th1 and Th2 effector molecules antagonize the development of Th17 cells, which are responsible for destructive tissue pathology in autoimmune diseases including neuroinflammation [24–26]. Whereas, naïve CD4 cells in the presence of IL-12 and transcription factors, such as T-bet and STAT4, become Th1 cells, which express IL-12R and produce IFN-*γ*. However, naïve CD4 cells in the presence of IL-4, GATA-3 and STAT6 become Th2 cells, which produce IL-4. Finally, naive CD4 cells in the presence of TGF-*β*, IL-23 and transcription factor ROR*γ*t become IL-17-producing Th17 cells.

**Figure 2:**
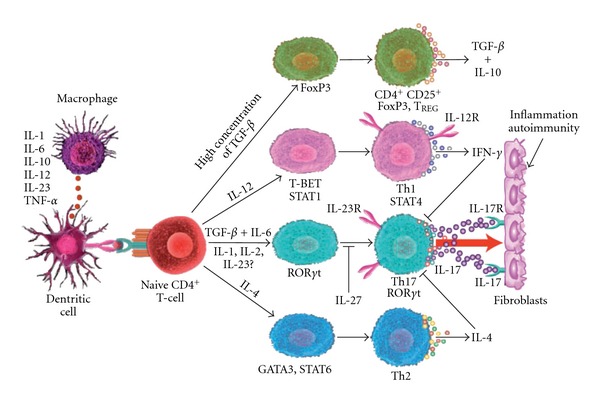
Cytokine production by dendritic cells and macrophages induce development of TReg, Th17 or IL-17-producing cells from naive CD4 cells and regulation of Th17 cells by Th1 and Th2 cytokines.

Playing on its pathogenic role, IL-17 produces an inflammatory cascade. IL-17 activates fibroblasts to produce proinflammatory cytokines such as TNF-*α*, IL-1*β* and IL-6, resulting in tissue inflammation and autoimmunity (Figure 2). From this figure it is possible to conclude that the level of immunoregulatory cytokine TGF-*β* is crucial for differentiation of Th17 cells. For CAM practitioners, these findings strongly suggest that targeting signature cytokine expression by Th1 or Th2 cells, in addition to targeting Th17 cells and their associated cytokines and transcription factors, directly or indirectly, may be more efficacious for the treatment of inflammatory and autoimmune disorders including neuroinflammation and neuroautoimmunity.

## 3. Th17 Development Requires TGF-*β* with IL-6

Recent studies demonstrate that the development of the human Th17 cell requires TGF-*β* and one or more proinflammatory cytokines such as IL-6 [27, 28]. Data generated in one study have shown that for Th17 development dendritic cells stimulated with *β*-glucan or zymosan, TGF-*β* and IL-6 were partially required, but the necessity for IL-1 was absolute [29, 30]. However, a different study in which naïve human CD4^+^ T cells were isolated from cord blood revealed that TGF-*β* plays a critical role in human Th17 differentiation [31]. In these and other studies it has been reported that very low concentrations of TGF-*β* such as 25 pg/mL, will give rise to Th17 cells, and high concentrations of TGF-*β* (2 ng/mL or more) will begin to favor production of regulatory T cells (CD4^+^ and CD25^+^), with or without the presence of IL-6 [32–41]. It could be concluded that regulation of T-helper cell differentiation can occur via coordinated activities of cytokines and transcription factors (see Figure 2). It seems that TGF-*β* produced by dendritic cells and macrophages can regulate both the differentiation to T_REG_s and IL-17-producing Th17 cells. Therefore, TGF-*β* induces co-expression of transcription factors FoxP3 and ROR*γ*t, which are important for differentiation of T_REG_ or Th17 cells, respectively. High concentrations of TGF-*β* (2 ng or greater) force the co-expression of FoxP3, which blocks ROR*γ*t-dependent gene expression, resulting in naïve CD4^+^ cell differentiation to T_REG_ cells. However, low concentrations of TGF-*β* (25 pg/mL or less) along with one of these proinflammatory cytokines, IL-6, IL-21, IL-23 or IL-1, induce the expression of ROR*γ*t resulting in the differentiation to Th17 positive cells and thus, the expression of IL-17 [7, 31, 32]. It seems that FoxP3 antagonizes ROR*γ*t via physical interaction, while inflammatory mediators, such as IL-6 and IL-21, relieve this inhibition possibly by a post-translational effect on either ROR*γ*t or FoxP3 [32, 42, 43].

## 4. Th17 Lymphocyte-Mediated Allergic Inflammation

It is accepted that CD4 T-helper lymphocytes are essential regulators of immune responses and inflammatory diseases [44]. Th2 cells mediate humoral immunity and allergic responses. In addition to Th2, Th17 cells and IL-17 have been implicated in allergic inflammation. This novel IL-17 cytokine family also mediates the migration and activation of inflammatory cells in the airways, resulting in inflammation that is typical in asthma [45, 46]. IL-17 has also been associated with many inflammatory and autoimmune diseases [47–49]. Recently, it was shown that IL-6 could regulate Th17 development in both mice and humans [50–52]. Therefore, it is not surprising that both Th2 and Th17 responses are elicited by different allergens in asthma [46]. In these conditions, IL-6 produced by dendritic cells (DCs) acts as a control regulator by not only promoting Th2 and Th17 responses, but also in limiting the Th1 response [50–52].

By producing IL-6, DCs are integral to differentiation of T-helper into Th2 and Th17 subsets. However, molecular mechanisms that regulate IL-6 production by DCs have not been elucidated. To identify a previously unknown signaling axis in DCs that inhibits Th1 immune response, but promotes the induction of Th2 and Th17 responses, experiments were performed using bacterial toxins and house dust mite (HDM) in both *in vitro* and *in vivo* systems [53]. Krishnamoorthy and colleagues [53] examined the multi-cellular pathway in which HDM (allergen) acts on dendritic cells to drive naïve CD4 cells to Th2 and Th17 cell differentiation. To clarify these molecular steps linking allergens' immunization to differentiation of T cells, they initially approached this problem by using microarray expression to identify genes induced by bacterial toxins and HDM. The authors incubated DCs either with cholera toxin or with HDM, the experimental allergen. Both bacterial toxin and HDM induced IL-6 secretion along with cell surface expression of C-KIT and its ligand, stem cell factor (SCF), on DCs, blocking IL-6 by monoclonal antibody prevented induction of both Th2 and Th17 cells. They also showed that this dual upregulation of C-KIT and SCF results in sustained signaling downstream of C-KIT, inducing IL-6 secretion. To show that both C-KIT and SCF are required for induction of IL-6 and activation of Th2 and Th17 cells, the researchers employed dendritic cells expressing mutants for C-KIT and SCF and cultured them with cholera toxin. Dendritic cells with compromised cell surface expression secreted less IL-6 [53].

**Figure 3:**
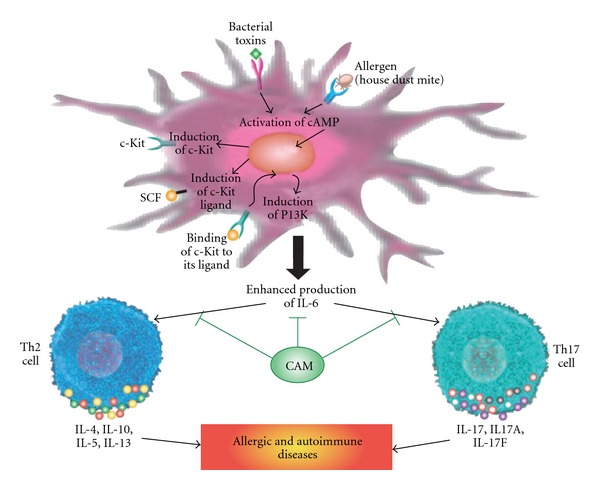
Effects of bacterial toxins and allergens on dendritic cell activation of cAMP induction of transmembrane signaling and its receptor ligand. Binding of c-Kit to its ligand SCF stimulates dendritic cells to secrete IL-6. IL-6 acts as a key factor in differentiation of T lymphocytes into Th2 and Th17 cells, which contribute to the development of allergic and autoimmune diseases. By inhibiting enhanced IL-6 production, CAM practitioners may prevent, slow down or reverse the development of allergic and inflammatory disorders.

The work of Krishnamoorthy and colleagues [53] shows that both bacterial toxins and allergens induce cell surface expression of C-KIT and its ligand SCF. The binding of C-KIT to its ligand activates PI3-kinase signaling, which stimulates the production of IL-6. Secreted IL-6 ultimately contributes to the induction of Th2 and Th17 cells, resulting in allergies and some autoimmune diseases (Figure 3). These findings suggest that the blocking of C-KIT binding to its ligand or inhibiting the production of IL-6 could be therapeutically useful for the treatment of allergic and autoimmune diseases. This approach is likely to stimulate further studies by CAM practitioners that could uncover additional targets for intervention in allergic and autoimmune diseases.

## 5. Contribution of Th17 Lymphocytes to Autoimmune Disorders

Immunocompetent cells in particular CD4^+^ T-lymphocytes are involved in the pathogenesis of chronic inflammatory disease by the production of unique sets of cytokines and cell surface molecules [54]. The activity of IFN-*γ*-producing Th1 lymphocytes has traditionally been linked to the induction and progression of tissue damage in *Psoriasis volgaris*, Crohn's disease, rheumatoid arthritis and other autoimmune diseases. Therefore, these diseases have been classified as Th1-associated disorders [5].

Recently, however, a novel subpopulation of memory CD4^+^ T-lymphocytes has been identified that produces high levels of IL-17, which plays a major role in the induction of inflammation and tissue destruction in various autoimmune disorders [55–60]. In addition, IL-17 is produced by *γδ* T cells, which are involved in collagen-induced arthritis (CIA) [61]. The depletion of *γδ* T cells during CIA results in less severe diseases, indicating a pathogenic role of these IL-17-producing cells [61]. IL-17 and its relatives IL-17A and IL-17F have very strong proinflammatory effects on many cellular targets, including fibroblasts, epithelial cells, endothelial cells, monocytes/macrophages, keratinocytes and osteoclasts [62]. To confirm the involvement of Th17 in inflammatory lesions, Pene and colleagues [62] isolated up to 30% of infiltrating lymphocytes from inflammatory lesions. These activated Th17 cells produced IL-26, TNF-*α*, lymphotoxin-B and IL-22. Produced at relatively high concentrations, these cytokines were inversely correlated with the production of Th1 and Th2 cytokines [63]. Results showed that tissue-infiltrating Th17 cells contribute significantly to human chronic inflammatory diseases through the production of inflammatory cytokines and create an environment that contributes to inflammatory diseases.

To support the pathogenic role of Th1, Th2 and Th17 in autoimmune diseases, Stummvoll and colleagues [64] compared the results of adoptively transferred Th1, Th2 and Th17 effector cells in the animal model of autoimmune gastritis induced by ATPase from gastric parietal cells as the target antigen. They found that while each injection of Th1, Th2 and Th17 cells induced gastritis, the transfer of Th17 effector cells caused the most dramatic pathology. At 4 weeks of cell injection, 20% of Th1 recipient, 60% of the Th2 recipient and 80% of Th17 recipient animals showed evidence of autoimmune gastritis. Interestingly, co-transfer of regulatory T cells (IL-10 and TGF-*β* secreting) was capable of reducing the incidence and severity of this autoimmune disease in Th1 and Th2 injected mice, but not in the Th17 injected group. Taken together, this strong inhibitory effect of T_REG_ on Th1 and Th2 effector cells makes T_REG_ an attractive CAM therapeutic target for some autoimmune diseases. However, if the autoimmune disease is caused by Th17 cells, it should then become an additional target for CAM treatment. This differentiation between Th1 and Th17 induction of autoimmunity requires laboratory measurement of these cytokines. Therefore, in assessing patients with autoimmune disorders, the measurement of Th1 (IFN-*γ*), Th2 (IL-4, IL-5, IL-13) and Th17 (IL-17) cytokine production by cultured lymphocytes will guide CAM practitioners in tailoring proper treatments for Th1- and Th17-mediated autoimmune diseases.

## 6. Induction of Central Nervous System Autoimmunity by Th17

IL-17-producing T-helper cells play an important role in the induction of autoimmune diseases, including multiple sclerosis and its animal model called experimental autoimmune encephalomyelitis (EAE) [65, 66]. This observation is based on the detection of IL-17 levels in both the plaques and cerebrospinal fluid of MS patients [67–69]. In the previous section, it was established that IL-17 is a proinflammatory cytokine that stimulates epithelial, fibroblast and endothelial cells to produce other inflammatory chemokines and cytokines, including macrophage inflammatory protein (MIP)-2, monocyte chemoattractant protein (MCP-1), granulocyte-colony stimulating factor (G-CSF) and IL-6 [47–49]. Interestingly, IL-17 synergizes with two inflammatory cytokines, IL-1*β* and TNF-*α*, to further induce chemokine expression [70, 71]. Since it is well known that microglia function as antigen presenting cells and effector cells and are involved in the inflammatory demyelination of the central nervous system (CNS), researchers examined the effect of IL-17 produced by Th17 cells on microglia in order to examine the contribution of IL-17 to inflammatory demyelination in the CNS. It was shown that treatment of microglia with IL-17 upregulated microglia production of IL-6, MIP-2, nitric oxide, neurotrophic factors and adhesion molecules. Also, when IL-1*β* and IL-23 were added to microglia, a significant amount of IL-17 was produced [72].

Microglia produce IL-1*β* and IL-23 under inflammatory conditions. These cytokines may act in an autocrine manner to further induce IL-17 expression in microglia, and thereby contribute to neuroimmune diseases, such as MS, in the central nervous system [72]. To further support the IL-17 association with neuroimmune disorders, mice injected with specific antibodies against IL-17 resulted in inhibition of chemokine expression, whereas overexpression of IL-17 in lung epithelia resulted in chemokine production and leukocyte infiltration. Thus, IL-17 expression characterizes a unique T-helper lineage, distinct from Th1 and Th2 cells, that plays a significant role in tissue inflammation [73].

In MS, the location of lesions in the CNS is variable and is a very important determinant of clinical outcome. This difference in lesion distribution, which is linked to HLA complex, suggests that T-cell specificity influences the sites of inflammation. Based on this observation, in a recent study it was demonstrated that T-cells specific to MBP epitopes generate two different populations of helper cells, Th17 and Th1 [74]. Notably, the Th17 to Th1 ratio of infiltrating T-cells determines whether or not inflammation occurs in the CNS. It was concluded that inflammation in the brain parenchyma occurs when the ratio of Th17 to Th1 is much greater than one. This outnumbering of Th1 by Th17 cells triggers a disproportionate increase in IL-17 in the brain resulting in inflammation. Consistent with the clinical phenotype, neutralization of IL-17 eliminated parenchymal inflammation in the brain [74]. Altogether, this study indicates that Th17, Th1 and their ratio, along with inflammatory cytokines, chemokines and adhesion molecules, are the mechanisms regulating cell infiltration into the brain parenchyma. Furthermore, the established differential regulation of inflammation in the brain with a Th17 : Th1 ratio >1, while in the spinal cord with a Th17 : Th1 ratio <1, indicates that Th1 cells play a significant pathologic role in spinal cord autoimmunity [74]. It was concluded that IL-17 produced by Th17 cells is the major regulator of central nervous system autoimmunity.

Production of IL-17 induces the activation of matrix metalloproteinase-3 (MMP-3) and recruits neutrophils to the site of inflammation. Neutrophil activation of enzymes such as MMPs, proteases and gelatinases, contributes to blood-brain barrier (BBB) breakdown. BBB breakdown further enhances the recruitment of neutrophils. This increase in protease activity, which attracts a significant number of monocytes and macrophages to the inflammatory sites, leads to sustained myelin and axonal damage [73, 75–77]. Together, these findings imply that CAM therapies, which may include medication, immune response modifiers or herbal medicine, that target IL-17 activity may be the most beneficial for patients with autoimmunities and neuroautoimmunities, particularly for patients with lesions in the brain.

## 7. Conclusion

On the basis of signature cytokine expression, transcription factors and signaling pathways, effector T-helper cells include Th1, Th2, Th3 and Th17. The latest T-helper to be defined, Th17, together with its cytokine family of IL-17, has been deemed the most pathogenic in inflammatory neuroimmune and autoimmune disorders. An activated T-cell, triggered by TGF-*β* in the presence of IL-6, differentiates to Th17. Subsequently, this T-helper then produces effector cytokines IL-17, IL-17A, IL-21, IL-22 and IL-26. This cytokine family is involved in inducing and mediating proinflammatory responses. Specifically, IL-17 mediates the migration and activation of inflammatory cells in the airways, resulting in the inflammation often seen in asthma, and is commonly associated with an allergic inflammatory response. Tissue-infiltrating Th17 cells contribute significantly to chronic inflammatory diseases. Inflammation in various tissues is achieved by secreted IL-17 and its relatives IL-17A and IL-17F due to their proinflammatory effects on cellular targets, which include endothelial cells, epithelial cells, fibroblasts, keratinocytes, monocytes/macrophages and osteoclasts. Under CNS inflammatory conditions, microglia, which act as antigen presenting cells, produce IL-1*β* and IL-23. Acting in an autocrine manner, these cytokines may further induce IL-17 expression in microglia, contributing to neuroimmune disorders. Another inflammatory pathway involving IL-17 is the IL-17-induced activation of MMP-3, which recruits neutrophils to the site of inflammation. Neutrophils then activate proteases and gelatinases, which contribute to an enhanced BBB permeability, and eventual myelin and axonal damage. Indeed, Th17 and IL-17 have far-reaching inflammatory properties. Thus, any patient who presents with autoimmune, inflammatory, or neuroimmune symptomatology would greatly benefit from an individualized treatment protocol. CAM practitioners may implement personalized therapies for these patients by measuring vital cytokine production that included IFN-*γ* (Th1), IL-4, IL-5 and IL-13 (Th2), and IL-17 (Th17). The modulation of IL-17, IL-6, NF-*κ*B and other factors may be useful therapeutic targets to CAM practitioners who tailor appropriate therapies for their patients exhibiting neuroimmune disorders. As with any research model, this work on CD4^+^ T-cell diversity in human diseases is a work in progress and is subject to enhancement in the coming years.
